# Technical feasibility and perioperative outcome of laparoscopic resection rectopexy with natural orifice specimen extraction (NOSE) and intracorporeal anastomosis (ICA)

**DOI:** 10.1007/s00423-022-02514-8

**Published:** 2022-04-28

**Authors:** Jamal Driouch, Omar Thaher, Ghaith Alnammous, Joachim Dehnst, Dirk Bausch, Torben Glatz

**Affiliations:** 1 Department of Surgery, Marien Hospital Herne, Ruhr-Universität Bochum, Hölkeskampring 40, 44625 Herne, Germany; 2 Department of Surgery, Paracelsus- Klinik Hemer, Breddestraße 22, 58675 Hemer, Germany

**Keywords:** Laparoscopic resection rectopexy, Obstructive defecation syndrome, Natural-orifice-specimen-extraction, Intracorporeal anastomosis

## Abstract

**Purpose:**

Laparoscopic rectosigmoid resection rectopexy (LRR) is the most effective treatment of obstructive defecation syndrome but is associated with a higher postoperative morbidity compared to transanal approaches. Natural orifice specimen extraction (NOSE) has been described as a promising technique to lower morbidity in colorectal cancer surgery. In this study, we analyze the technical challenges of adapting this technique to LRR and compare the perioperative results to the conventional laparoscopic technique with specimen extraction via minilaparotomy and extracorporeal anastomosis.

**Methods:**

We retrospectively analyzed 45 patients who underwent laparoscopic rectosigmoid resection rectopexy due to obstructive defecation syndrome at our institutions. From September 2020 to July 2021, we treated 17 consecutive patients with NOSE-LRR and compared the results to a historic cohort of 28 consecutive patients treated with conventional laparoscopic rectosigmoid resection rectopexy plus minilaparotomy (LAP-LRR) for specimen extraction between January 2019 and July 2020. Assessed were patient- and disease-specific parameters, operative time, hospital and postoperative complications and subjective patient satisfaction after 6 months of follow-up.

**Results:**

Both groups were comparable in terms of gender distribution, age, and comorbidities. The median operating time was similar and the perioperative morbidity was comparable in both groups. The length of stay in hospital was significantly shorter in the NOSE-LRR group (median 6 vs 8 days).

**Conclusion:**

NOSE-LRR can be implemented safely, performed in a comparable operating time, and is associated with a comparable rate of postoperative complications. The technique offers the a potentially fast postoperative recovery compared to the conventional laparoscopic technique.

## Introduction

Obstructive defecation syndrome (ODS) is a common disorder in the western population and is associated with severe impairment of quality of life. Morphological alterations of the colorectum and pelvic floor lead to a functional disorder with obstructive outlet, frequently accompanied by incontinence and constipation [1]. The extent of the dysfunction has to be thoroughly determined by a precise anamnesis and disease-specific assessment systems, as well as a clinical proctological examination, including rectal inspection, digital rectal examination, proctoscopy and rectoscopy and, in many cases, clinical imaging such as endosonography, contrast enema and dynamic defecography, and possibly MR-defecography.

While morphological changes of the pelvic floor alone without impairing clinical symptoms do not constitute an indication for surgery [2], several effective methods for the surgical treatment of ODS have been established for symptomatic patients. Up to date, a standardized pathway for the operative care of ODS does not exist [3]. Transabdominal techniques and the transanal stapler resection have been established side by side and both have their place in proctological surgery today. Surgical techniques used in the treatment of ODS include laparoscopic resection rectopexy, Delorme’s procedure, laparoscopic suture rectopexy, laparoscopic mesh rectopexy, and stapled transanal rectal resection (STARR/ Trans-STARR) [4–9]. All of these techniques have either a proximal or a distal outlet obstruction. However, many patients who become symptomatic have a combination of both proximal and distal outlet obstruction. In the attempt to target both the proximal outlet obstruction (rectosigmoid resection) and the distal obstruction (suture rectopexy) in one surgery, the rectosigmoid resection rectopexy has been developed [10, 11]. Established indications for laparoscopic resection rectopexy are sigmoid resection for dolichosigmoid with sigmoidocele, cul de sac syndrome, external rectal prolapse, or sigmoid stricture with advanced intussusception [10].

While transabdominal approaches generally offer more sustainable options for pelvic floor repair, they are associated with a higher risk of intra- and postoperative complications compared to transanal approaches.

Laparoscopic approaches combined with natural orifice specimen extraction (NOSE) have become increasingly popular over the last decade and proved to be superior to conventional laparoscopy with minilaparotomy in randomized controlled trials, with fewer complications, shorter hospital stay, decreased postoperative pain and faster recovery [12, 13]. The technique has been adapted in specialized centers for colorectal resections due to cancer or diverticulitis and proved to be safe and beneficial for the patients [14–18].

The potential benefits of NOSE, including reduced postoperative pain and wound complications, reduced use of postoperative analgesics, faster recovery of bowel function, shorter length of hospital stay, and better cosmetic and psychological outcomes, have been demonstrated in colorectal surgery. Avoidance of extraction site laparotomy and a lower incisional hernia rate is the most important features of NOSE [19].

The implementation of NOSE into transabdominal pelvic floor repair techniques could potentially offer patients the benefit of definitive abdominal repair without the cost of additional morbidity, restricting perineal procedures to high-risk or the very elderly patients [20].

## Methods

This retrospective cohort study included 45 patients who underwent laparoscopic surgery for ODS between January 2019 and July 2021. We performed laparoscopic rectosigmoid resection rectopexy with ventral peritoneal suture pexy. Criteria for laparoscopic rectosigmoid resection rectopexy were dolichocolon with cul de sac syndrome/sigmoidocele, sigmoid kinking stenosis with pronounced intussusception II–III, rectal prolapse I–III, or a combination of dolichocolon with intussusception and rectal prolapse and descending perineum syndrome. Exclusion criteria were slow transit obstruction, irritable bowel syndrome, severe comorbidity with highly elevated risk for general anesthesia, previous sigmoid resection, or previous pelvic radiation. Previous abdominal and pelvic surgery, both minimally invasive and conventional, was a contraindication to laparoscopic rectosigmoid resection only when laparoscopic access to the abdominal cavity was considered unlikely.

Up until August 2020, patients underwent laparoscopic surgery with a minilaparotomy for specimen extraction and insertion of the stapler anvil (either suprapubic or in the left lower abdomen) (28 patients, LAP-LRR). As of September 2020, the technique was modified by the omission minilaparotomy and transanal extraction of the specimen (17 patients, NOSE-LRR). The anastomosis was created intracorporeally (ICA). Endpoints of the study were the operating time, length of hospital stay, and postoperative complication rate according to Clavien-Dindo (including anastomotic stenosis/leak, abdominal or endoluminal hemorrhage, and wound infection). Due to the retrospective study design, exact pain scores were not available for this cohort.

### Diagnostic work up and patient follow-up

The indication for surgery was clinical complaints with typical symptoms and clinical findings. Since not all patients with proctological complaints require extensive coloproctological diagnostics, we have established criteria for extended diagnostics: patients with subjective incomplete defecation or unsuccessful defecation associated with proctoscopic/rectoscopic findings of intussusception II–III as well as rectal prolapse and descending of the pelvic floor routinely receive an extended diagnostic work-up. This includes endosonography, contrast enema, and dynamic imaging such as X-ray defecography or MR defecography and measurement of colon transit time. Anal manometry was performed to rule out sphincter defects in patients with incontinence.

Patients were followed up regularly in our outpatient clinic at 1, 3, and 6 months after surgery, including protologic examinations and disease-specific assessment systems.

### Operative technique

The patient underwent standard bowel preparation on the day before surgery. *A selective intestinal decontamination was not routinely used*. An enema was administered directly preoperatively. All procedures were performed 30 min prior to incision under general anesthesia in the Lloyd-Davies position with antibiotic prophylaxis. The patient was immobilized on the table with neck supports or a vacuum mattress. After insertion of a transurethral catheter and standard abdominal preparation, a semi-sterile rectoscopy was performed to assess mucosal perfusion and rule out rectal stenosis or kinking and to determine the planned stapler size.

Trocars were placed as follows: a 5-mm trocar between the medio clavicular axis and the anterior axillary line 2–3 cm distal to the last rib, a 12-mm trocar in the right lower abdomen 2–3 cm medial to the anterior superior iliac spine, and a 12-mm camera trocar 2 cm above the navel. In the LAP-LRR group, a 5-mm auxiliary trocar was rarely placed at the sampling site. A 5-mm auxiliary trocar was rarely placed at the recovery site in the LAP-LRR group. After inflation of the capnoperitoneum (12–14 mmHG), peritoneal adhesions from previous operations were released if necessary. The pelvic preparation was performed in a 16° Trendelenburg position with a 10° tilt to the right. The descendorectostomy was created transanal as an end-to-end anastomosis with a circular stapler (size 28, 29, or 31 mm, as required).

### Surgical steps

The peritoneum of the rectosigmoid is incised medially at the level of the promontory in a caudal direction along the rectum, followed by blunt dissection into the Waldeyer layer. The autonomic nerves remain dorsal, while the dorsal dissection continues to the coccygeum. The lateral paraprocts are cauterized. The left ureter is visualized from the medial or lateral side. On the lateral side, the Toldt fascia is incised, sparing the ureter, and the sigmoid is mobilized to the branch of the inferior mesenteric artery to the branch of the descending colon and the superior rectal artery. Starting from the promontory, the tubular mesosigmoid is divided cranially to the proximal resection border. This exact resection line is finally established during transanal extraction. The superior rectal artery is dissected distally.

Ventrally, the peritoneum is incised approximately 1 cm distal to the peritoneal fold and the Denonvillier layer is dissected over approximately 3 cm. The rectum is stretched cranially, followed by anal inspection to identify prolapse.

In the LAP-LRR group, the mesorectum of the medial rectum is skeletonized and the rectum is dissected with an articulated linear stapler. After a mediolateral incision in the lower abdomen, a protective sheet is inserted over which the specimen is extracted. After applying a purse-string suture at the proximal resection border and sharp resection of the specimen, the stapler anvil is fixed and then reinserted into the abdomen. The insertion is closed, and the pneumoperitoneum is reinflated.

**Fig. 1:**
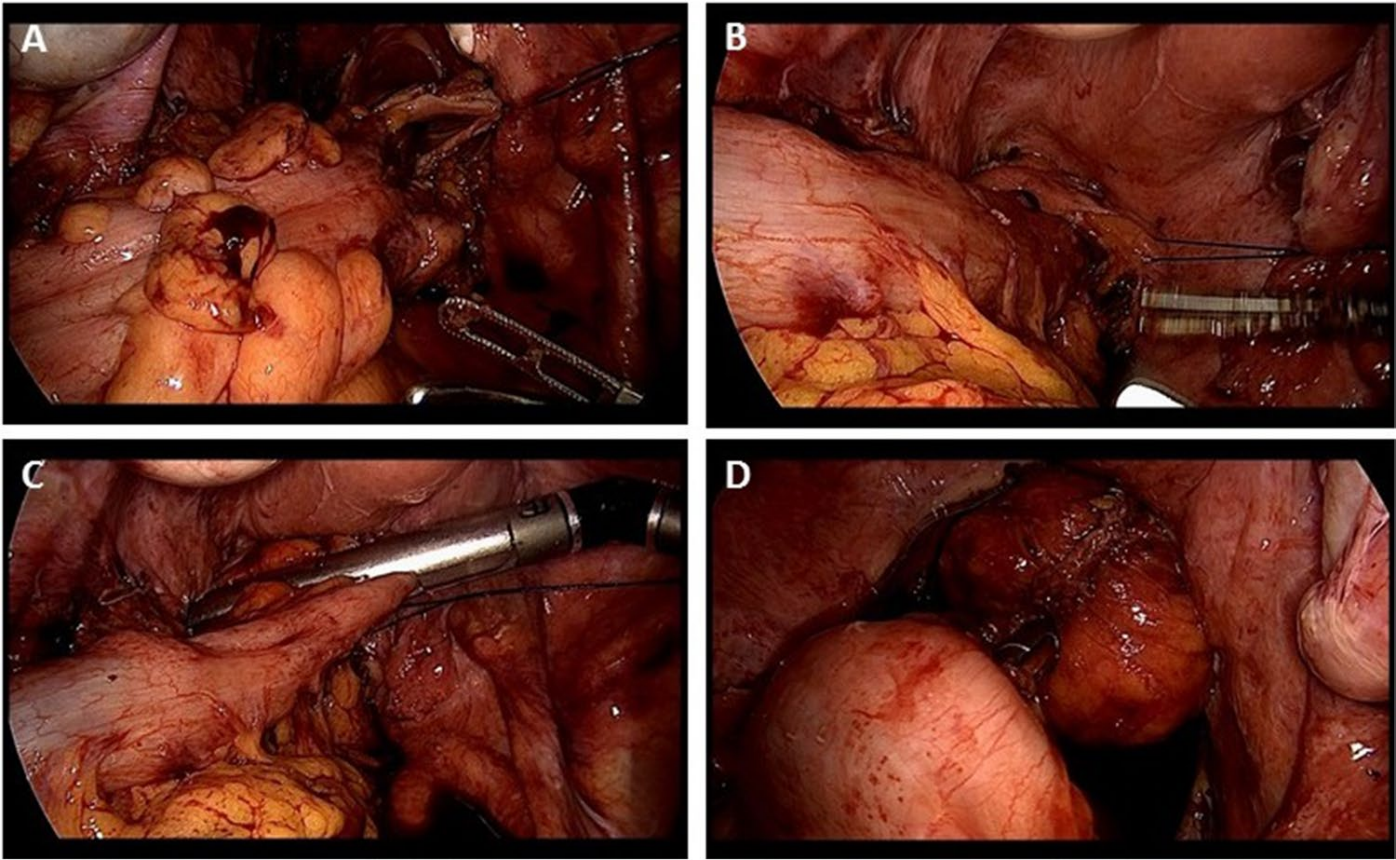
Transanal specimen extraction and intracorporeal preparation of the descendorectostomy. **A** The rectum is dissected with monopolar scissors and the lumen exposed with an additional holding thread. **B** The specimen is extracted transanally. **C** After transanal insertion of the anvil, the descending colon is dissected with an angled linear stapler. **D** The anvil is extracted at the suture line and the anastomosis is created with the transanally introduced circular stapler

In the NOSE-LRR group, the rectum is cut 3 cm proximal to the resection border with monopolar scissors. A Vicryl thread is attached laterally to keep the intestinal lumen open. Transanally, the severed end of the rectum is grasped with a forceps and recovered from the rectum. The proximal resection line can now be determined precisely. The stapler anvil with tip and thread is pushed forward through the intestinal lumen to the proximal point of the resection line. The bowel is dissected distally of the anvil with an articulated linear stapler. The specimen is removed. Subsequently the rectal stump is dissected with an additional linear stapler row at the level of the skeletonized mesorectum (approx. 3 cm) and recovered in a retrieval bag over the 12-mm trocar. Now the anvil must be pulled through the staple row sealing the descending colon. The attached thread is grasped, and the anvil is pressed bluntly through the staple suture, or a small hole is sharply incised, and the anvil is passed through. The tip is removed and recovered over the 12-mm trocar (Fig. 1).

Subsequently, in both groups, the circular stapler is introduced transanally, and the tip is extended and connected to the anvil. After creating the anastomosis, a leak test by air insufflation is performed. Our strategy for positive air leak test is usually placement of additional laparoscopic sutures at the leak and repetition of the test. If sutures failed, placement of a diverting ileostomy or resection and recreation of the anastomosis is discussed individually.

The peritoneal fold is doubled with a continuous barbed suture and 3 single sutures on the ventral rectal wall at the anastomotic site. A Robinson drainage is placed in the pelvis and diverted to the right. Supraumbilical fascia closure is performed. In the LAP-LRR group, the peritoneum of the extraction incision is continuously closed with Vicryl and the fascia with a PDS suture. A Redon drainage is inserted in patients with an obese subcutaneous fat layer > 3 cm or other risk factors for postoperative wound infections.

### Statistical analysis

Statistical analysis was performed using IBM SPSS statistics, version 27. Categorical variables were put in absolute and relative frequencies; differences were evaluated by chi-squared or Fisher’s exact test as appropriate. Quantitative values were expressed as medians with range, and differences were measured using the Mann–Whitney *U*-test. A *p*-value < 0.05 was considered statistically significant.

## Results

### Patient characteristics

The patients included were between 26 and 94 years old at the time of the operation. The median age was 63 years. The majority of patients were female (*n* = 35 (78%)). A total of 28 patients received a LAP-LRR procedure, while NOSE-LRR was performed in 17 patients. Patient characteristics were evenly balanced among the two groups (Table 1).

**Table 1:** Demographic data

	LAP-LRR (*n* = 28)	NOSE-LRR (*n* = 17)	All (*n* = 45)	*p*
Gender Male Female	7 (25%) 21 (75%)	3 (18%) 14 (82%)	10 (22%) 35 (78%)	0.426
Age in years *(median)	56.5 (range 26–91)	69 (range 31–94)	63 (range 26–94)	0.106
Obstipation	25 (89%)	15 (88%)	40 (89%)	0.635
ASA I II III	4 (14%) 14 (50%) 10 (36%)	2 (12%) 11 (65%) 4 (24%)	6 (13.3%) 25 (56%) 14 (31.1%)	0.629
BMI (median)	27 (range 20.7–35.9)	26 (range 17.9–34.6)	26 (range 17.9–35.9)	0.361
Previous abd./rect. op`s 0 1–2 ≥ 3	4 (14%) 16 (57%) 8 (29%)	4 (24%) 5 (29.4%) 8 (47%)	8 (18%) 21 (47%) 16 (36%)	0.675

Four (14%) patients in the LAP-LRR group and four (24%) in the NOSE-LRR group had no history of previous abdominal or pelvic surgery, while 16 (57%) patients in the LAP-LRR group and 5 (29%) in the NOSE-LRR group had up to 2 abdominal surgeries and 8 (29%) patients in the LAP-LRR and 8 (47%) in the NOSE-LRR group had 3 or more abdominal or rectoanal surgeries (*p* = 0.675). There were also no significant differences with regard to the ASA score. Four (14%) patients in the LAP-LRR group and 2 (12%) in the NOSE-LRR group had an ASA score of 1. Fourteen (50%) patients in the LAP-LRR group and 11 (65%) in the NOSE-LRR group had an ASA score of 2. Ten (36%) patients in the LAP-LRR group and 4 in the NOSE-LRR group (24%) had an ASA score of 3 (*p* = 0.629). The median BMI in the LAP-LRR group was 27 and tended to be slightly higher than in the NOSE-LRR group with 26 (*p* = 0.361). Patients’ characteristics are displayed in Table 1.

25 (89%) patients in the LAP-LRR group had constipation preoperatively, comparable to the NOSE-LRR group (15 (88%) *p* = 0.635). In the LAP-LRR group, a rectocele was found in 12 (43%) patients and in the NOSE-LRR group in 12 (71%) patients (*p* = 0.066).

### Surgical results

Operating time was 120 min (median) in the LAP-LRR group and 130 min (median) in the NOSE-LRR group with no statistically significance (*p* = 0.558). There was no conversion from laparoscopic to open surgery in either group. Postoperative complications occurred with a comparable frequency in both groups (*p* = 0.82), but specific procedure associated complication varied slightly among the groups.

Postoperative wound infections occurred strictly in the LAP-LRR group in a very low frequency (2 versus 0 in the NOSE-LRR group; *p* = 0. 382). No incisional hernias were noted in the postoperative follow-up of 6 months.

Intraoperatively fashioning of the anastomosis was uncomplicated in all cases without any positive air-leak test. However, in the LAP-LRR group, an anastomotic leak occurred after 21 days; the same patient suffered ureteral injury with formation of a urinoma during primary surgery. There was also an anastomotic leak in the NOSE-LRR group, which was diagnosed on the 3rd postoperative day and reoperated in a rendezvous procedure by transanal minimally invasive suture and laparoscopic transabdominal drainage plus diverting enterostomy.

Endoluminal anastomotic hemorrhage occurred in two patients in the NOSE-LRR group immediately postoperatively and was clipped endoscopically on the same day, whereas in the LAP-LRR group, no endoluminal anastomotic hemorrhage occurred. Two patients in the LAP-LRR group suffered from postoperative intrabdominal hemorrhage, which did not occur in the NOSE-LRR group (*p* = 0.244).

Perioperative complications were evaluated using the Clavien-Dindo classification. Complications were more frequent and more severe in the LAP-LRR group without being statistically significant (*p* = 0.494). In the LAP-LRR group, 13 (46%) patients had no complications, 7 (25%) patients were treated with pain medication due to wound pain in an outpatient setting (Clavien II), and 2 (7%) had an anastomotic stenosis, which could be endoscopically dilated (Clavien IIIa). And 5 (18%) were categorized as Clavien III b. Two patients required surgical revision of the extraction incision due to scar pain, one anastomotic stenosis required surgical revision, and two patients were reoperated due to intraabdominal hemorrhage. The patient with anastomotic leak and ureteral injury was classified as Clavien IVa. In the NOSE-LRR group, 11 patients (65%) had no complications, 3 patients (18%) required prolonged pain medication due to wound pain (Clavien II), and in 2 (12%) patients, endoluminal anastomotic hemorrhage was managed endoscopically (Clavien IIIa). One patient with anastomotic leak was reoperated (Clavien IIIb).

The median length of stay in hospital was 6 days in the NOSE-LRR group and was significantly shorter than in the LAP-LRR group with 8 days (*p* < 0.001). Patients were questioned postoperatively regarding their subjective satisfaction with the result of the operation, which was comparable in both groups. 22 (79%) patients in the LAP-LRR group and 14 (82%) patients in the NOSE-LRR were satisfied with the surgical results and postoperative recovery (*p* = 0.538) (Table 2).

**Table 2:** Comparison of LAP-LRR and NOSE-LRR

	LAP-LRR group (*n* = 28)	NOSE-LRR group (*n* = 17)	All (*n* = 45)	*P*
Hospital stay (d)	8 (range 7–16)	6 (range 3–13)	8 (range 3–16)	0.000
Operating time in minutes (median)	120 (range 75–169)	130 (90–197)	121 (75–197)	0.558
Pain/nausea	9 (32%)	3 (18%)	12 (27%)	0.239
Wound healing dist	2 (7%)	0 (0%)	2 (4%)	0.382
Clavien-Dindo 0/I II IIIa IIIb IVa	13 (46%) 7 (25%) 2 (7%) 5 (18%) 1 (4%)	11 (64.7%) 3 (18%) 2 (12%) 1 (6%) 0	24 (53%) 10 (22%) 4 (9%) 6 (13%) 1 (2%)	0.494
Anastomotic leak	1 (4%)	1 (6%)	2 (4%)	0.618
Anastomotic stenosis	3 (11%)	0 (0%)	3 (7%)	0.231
Postoperative hemorrhage Abdominal Anal	2 (7%) 0 (0%)	0 (0%) 2 (12%)	2 (4%) 2 (4%)	0.104
Reoperation	6 (21%)	1 (6%)	7 (16%)	0.167
Subjective satisfaction	22 (79%)	14 (82%)	36 (80%)	0.538

## Discussion

In this study, we were able to demonstrate the feasibility and safety of our modified method of laparoscopic rectosigmoid resection with natural orifice specimen extraction (NOSE) and intracorporeal anastomosis (ICA).

### Adaption of the surgical technique

For an experienced surgeon, the preparation in the small pelvis while protecting the autonomic nerves is possible to master after an initial learning curve (LC) of approximately 50 to70 cases [12, 21]. The preparation technique was not modified in the NOSE-LRR group. No published data on the LC for NOSE-LRR is available: Data for the LC for robotic NOSE-procedures in colorectal surgery varied from 15 to 42 cases [22]. We routinely used only three trocars for the patients operated without laparotomy. It can be particular challenging to perform this procedure with only three trocars; however, addition of a fourth trocar is possible and will most likely not affect patients’ outcome and satisfaction. The difficulty of the NOSE-technique is also increased due to the fact, that the rectal stump has to be closed by the articulated stapler after the specimen has been removed transanally. The articulated stapler is often uncomfortable in the small pelvis and is difficult to maneuver. Particular skill is required here. In our experience, difficulties arise in the case of a stenotic lumen of the rectal-sigmoid junction, as the anvil has to be passed through here. In all cases to date, we have been able to successfully dilate the colon to make it passable for the anvil. The surgical procedure is delayed on average by 10 min by the transanal specimen extraction and ICA. A review of the literature confirms that NOSE has a longer operating time [16, 23, 24]. The insertion of the stapler anvil though the sigmoid transanally tends to be more time consuming compared to the minilaparotomy technique. Another factor is the extraction of the anvil: After insertion of the anvil in the descending colon, the tip of the anvil must be carefully extracted through the row or next to the row of staples with only 2 working instruments.

Already in 1993, Franklin et al. [25] described first clinical series of laparoscopic procedures for a variety of colonic lesions after developing basic techniques in animal models with an acceptable rate of complications and survival for malignant and nonmalignant disease processes. In the following period, laparoscopic colon resection was routinely established in many hospitals, and initial NOSE techniques were developed, but no statistically significant difference or advantage over laparoscopically assisted procedures were demonstrated [26]. In the past, several techniques were developed for NOSE. A study of 20 matched female patients undergoing transvaginal hybrid-NOTES sigmoidectomy or traditional laparoscopic sigmoidectomy for diverticulitis showed no significant differences in the sum of pain levels, length of procedure, intra-, and postoperative complications, but significant positive effects of the NOTES technique regarding morphine requirement postoperatively and length of hospital stay [27]. The main advantage of transvaginal NOSE is the possibility to extract large specimens from both right-sided and left-sided colonic resections, but this approach is only applicable in selected female patients [19].

In the development of the NOTES technique, many groups started by transvaginal techniques and developed trananal techniques from there. Bulian described 139 colonic resections between October 2008 and January 2013, using data from the German NOTES Registry. A total of 87% were sigmoid resections. A total of 88% were conducted transvaginally and only 12% transrectally [28]. Since transrectal NOSE was not yet standardized, it was tried in high selected patients (52%) using the NOSE technique [29]. Fuchs et al. investigated the implementation of laparoscopic sigmoid resection to the NOTES techniques with transanal extraction and, in addition to similar difficulties, concluded that transanal colon resection was a practicable and safe NOTES procedure [30].

Transanal specimen extraction is used for low anterior resection of the rectum with total mesorectal excision, when a coloanal anastomosis is planned [31]. Robotic intracorporeal surgery for complicated diverticulitis resection with natural-orifice intracorporeal anastomosis has been described by using a seven key technical modification [17]. We have developed a surgical NOSE-technique that allows the TME and anastomosis at every level of the rectum. While we release the rectum in the TME layer and divide it in the middle third, the robotic procedure divides the mesentery close to the bowel until the upper third of the rectum is reached. In preparation for the ICA, they introduce the circular stapler device transrectally. The anvil is detached, introduced, and then secured to the open left colon with a purse string suture, the rectal cuff is closed around the spike of the stapler with a second purse string suture, and the colorectal anastomosis is performed. Laparoscopic NOSE is also used for low/ultralow anterior colorectal cancer resection, using the prolapse technique [14–16, 18].

### Surgical outcome for NOSE-LRR

Our study demonstrates a superiority of the NOSE group with regard to hospital stay (6 days versus 8 days; *p* < 0.001). The average hospitalization after laparoscopic colectomy ranges between 5 and 9 days in randomized controlled trials [16, 23, 32]. The operating time in this study is slightly lower in comparison to the published mean operating time for NOSE (130 min vs 165–223.9 min) [33, 34] and (120 min vs 144 min) for LAP-LRR [10].

Regarding postoperative complications, we were able to identify noticeable differences among the two groups in frequency and type of postoperative complications but failed to demonstrate any significant distinction possibly due to the small cohort studied. Postoperative pain was reported more frequently in the LAP-LRR group (32% vs 18%) while an operative scar revision had to be performed in 2 patients because of scar pain. These findings are more common than in other studies [13, 16, 23]. This complication could not occur in the NOSE group due to the omission of an incision. In total, 6 patients required reoperation in the LAP-LRR group, whereas only 1 patient required surgical revision in the NOSE-LRR group. The results match the results of other studies [15, 16, 21, 23]. A 11% vs 0% anastomotic stenosis occurred in the LAP-LRR group (*p* = 0.231). The tension-free anastomosis in the NOSE-LRR group may have been carried out more precisely because the staple resection of the descending colon is performed intra-abdominally and the intestine is placed precisely at the position of the anastomosis during the staple transection. In the literature, similar frequencies have been recorded [32]. One anastomotic leakage occurred in both groups, which, despite the occurrence at 21 and at 3 days, was probably due to surgical technical errors. Similarly low anastomotic leakages are also reported by other studies with focus on postoperative outome after NOSE-procedures [33, 35]. Intra-abdominal hemorrhage occurred more frequently and endoluminal bleeding less frequently in the LAP-LRR group. This seems to be coincidental, as the surgical preparation technique and mechanical anastomosis installation are similar and do not explain this discrepancy. However, the additional manipulation at the rectal stump due to the specimen extraction could possibly explain the higher rate of endoluminal hemorrhage in the NOSE group. Other groups have described a significant lower blood-loss during NOTES-procedures, matching our findings [35, 36]; however, data on postoperative endoluminal hemorrhage after NOTES-procedures compared to conventional laparoscopy was not available in the literature.

With minor technical adaptations, our surgical technique can also be utilized for cases of malignant intestinal diseases of the left hemicolon up to the rectum. Potential pitfalls of the NOSE-procedure are bacteriological contamination of the peritoneal cavity [37, 38]. In order to solve this problem, prophylactic antibiotics, mechanical bowel preparation, intraoperative peritoneal irrigation, intraoperative transanal lavage with povidone–iodine, and normal saline, the use of transluminal wound retractor and placement of pelvic or abdominal drains have been recommended to reduce the bacterial contamination during the procedure [19, 37, 39]. A selective intestinal decontamination would be another option to reduce bacterial decontamination. When adapting the NOSE-technique for oncological procedures, another major concern remains, regarding the oncological safety. Tumor-related manipulation mainly arises from specimen extraction via narrow natural orifice, with the potential for compromise in oncological safety [40]. Furthermore, the local recurrence rate and long-term oncologic results 3-year recurrence rates after NOSES are comparable with conventional laparoscopic surgery [35].

## Conclusion

Laparoscopic resection rectopexy with natural orifice specimen extraction is technical feasible and can be adopted by an experienced colorectal surgeon without significantly prolonged operating times. The procedure is safe with a low complication rate and potentially superior to conventional laparoscopic resection rectopexy in terms of postoperative pain, wound infections and hospital stay.

## Data Availability

The original data is available on request.
